# Associations Between Retinal Vascular Occlusions and Dementia

**DOI:** 10.3390/healthcare12232371

**Published:** 2024-11-26

**Authors:** Minali Prasad, Deniz Goodman, Sanhit Gutta, Zahra Sheikh, Howard J. Cabral, Jenny Shunyakova, Nayan Sanjiv, Cameron Curley, Rohun Reddy Yarala, Lynna Tsai, Nicole H. Siegel, Xuejing Chen, Vasiliki Poulaki, Michael L. Alosco, Thor D. Stein, Steven Ness, Manju L. Subramanian

**Affiliations:** 1 Department of Ophthalmology, Boston University Chobanian & Avedisian School of Medicine, Boston, MA 02118, USA; 2 Department of Ophthalmology, Boston Medical Center, Boston, MA 02118, USA; 3 Department of Biostatistics, Boston University School of Public Health, Boston, MA 02118, USA; 4 VA Boston Healthcare System, Boston, MA 02130, USA; 5 Boston University Alzheimer’s Disease Research Center, Boston University Chobanian & Avedisian School of Medicine, Boston, MA 02118, USA; 6 Boston University CTE Center, Boston University Chobanian & Avedisian School of Medicine, Boston, MA 02118, USA; 7 Department of Neurology, Boston University Chobanian & Avedisian School of Medicine, Boston, MA 02118, USA; 8 Department of Pathology and Laboratory Medicine, Boston Medical Center, Boston University Chobanian & Avedisian School of Medicine, Boston, MA 02118, USA; 9 VA Bedford Healthcare System, Bedford, MA 01730, USA

**Keywords:** retinal vein occlusion, retinal artery occlusion, Alzheimer’s disease, vascular dementia

## Abstract

Background/Objectives: Retinal vascular occlusions, such as retinal vein occlusion (RVO) and retinal artery occlusion (RAO), are associated with cognitive impairment, including dementia. Our objective was to examine the odds of dementia among patients with retinal vascular occlusion. Methods: This cross-sectional study included 474 patients with retinal vascular occlusion and 948 patients without retinal vascular occlusion (comparison group). Patients in the comparison group were age- and sex-matched to those with vascular occlusion. Logistic regression was used to analyze the odds of all-cause dementia, vascular dementia, and Alzheimer’s disease after adjusting for demographic, clinical, and ophthalmic covariates. Main outcome measures included the presence of all-cause dementia, vascular dementia, and Alzheimer’s disease. Results: Patients with RVO (n = 413) had increased odds for all-cause dementia (odds ratio (OR) = 2.32; 95% confidence interval (CI): 1.44–3.75; *p* < 0.001) and vascular dementia (OR = 3.29; 95% CI: 1.41–7.68; *p* = 0.006) relative to the comparison group. Patients with central RVO (n = 192) (OR = 2.32; 95% CI: 1.19–4.54; *p* = 0.014) or branch RVO (n = 221) (OR = 2.68; 95% CI: 1.30–5.50; *p* = 0.007) had increased odds for all-cause dementia relative to the comparison group. Patients with RAO (n = 61) did not have increased odds of all-cause dementia (OR = 1.01; 95% CI: 0.32–3.26; *p* = 0.983), vascular dementia (OR = 1.54; 95% CI: 0.22–10.81; *p* = 0.663), or Alzheimer’s disease (OR = 0.32; 95% CI: 0.05–2.20; *p* = 0.244). Conclusions: A history of any RVO is associated with increased rates of all-cause dementia and vascular dementia independent of shared cardiovascular risk factors. These associations are not seen with a history of RAO, or between any subtype of vascular occlusions and Alzheimer’s disease.

## 1. Introduction

Dementia is a syndrome defined by the presence of cognitive and functional impairment and affects more than 55 million individuals across the globe [1]. The prevalence of dementia is projected to increase to 78 million by 2030 and 139 million by 2050 according to World Health Organization estimates [1]. The estimated global economic burden of dementia is anticipated to reach nearly USD 2.8 trillion by 2030 [1]. These projections of disease burden compel the need for early screening, detection, diagnosis, and the identification of risk factors in order to initiate therapeutics during early stages to prevent the progression of the disease. The underlying causes of the dementia subtypes, namely, Alzheimer’s disease (AD) and vascular disease (VD), are known to be multifactorial; however, their commonalities include microvascular changes in the cerebral circulation, cerebrovascular injury, and neuroinflammation [2]. Unmodifiable risk factors associated with the development of dementia include age, family history, and genetic susceptibility [3]. However, there is a growing body of literature suggesting the targeting of modifiable cardiovascular, lifestyle, and social risk factors as preventative interventions [4].

The retina is an extension of the central nervous system and shares embryological origins with the brain [5,6,7]. Diseases of the eye such as diabetic retinopathy, age-related macular degeneration, and glaucoma have been associated with neurodegenerative diseases such as Alzheimer’s disease, Parkinson’s disease, multiple sclerosis, and amyotrophic lateral sclerosis, indicating that those with eye disease are at risk of developing dementia [8,9,10,11,12,13,14,15].

Recently published large population-based cohort studies identified a link between dementia and retinal vascular occlusions [16,17,18,19,20]. These prior studies were based on International Classification of Diseases (ICD)-9 and ICD-10 codes, and some of these studies did not differentiate between retinal vein and artery occlusions, each of which has different pathophysiological mechanisms despite shared cardiovascular risk factors [21]. RAO occurs from cardiac emboli, vasculitis, spasms, and other arterial blockages in blood flow to the eye [21,22]. In contrast, RVOs are due to a thrombus occlusion near the lamina cribrosa (CRVO) or at an arteriovenous crossing point (BRVO) in a retinal vein [21]. The global incidence of any RVO is 0.86% and that of RAO is approximately 0.001% [23,24].

In this study, we performed a retrospective medical record review to examine the demographic and medical factors associated with retinal vascular occlusions and dementia among a diverse population of patients to further explore the relationship between these two disorders. In contrast to prior studies, we evaluated the potential association between dementia and subtypes of RVO (branch versus central) in separate analyses. Furthermore, we confirmed ICD-9 and ICD-10 diagnoses with a record review, unlike in large population-based cohort studies.

## 2. Materials and Methods

### 2.1. Study Population

This is a single-center, retrospective cross-sectional study that was approved by the Boston University Medical Center Institutional Review Board (IRB) and adheres to the tenets of the Declaration of Helsinki. The IRB granted a waiver of HIPAA Authorization for Research under 45 CFR164.512 (i) (2) (ii). This study was conducted at Boston Medical Center, an academic, urban-based safety net hospital.

We completed a retrospective review of electronic medical records of patients with retinal vascular occlusion and corresponding age- and sex-matched patients without vascular occlusions (comparison group) seen in the eye clinic. A 1:2 ratio of patients with and without retinal vascular occlusion was used to increase the statistical power [25]. We included patients who were 50 years of age or older with a diagnosis of any retinal vein occlusion (RVO) or any retinal artery occlusion (RAO) between September 2015 and November 2022. We did not include patients younger than 50 years of age due to differences in pathological mechanisms in the development of RVO between older and younger patients [26]. Given that dementia is strongly associated with older age and female sex [27,28], the comparison group was sex- and age-matched (within 3 years) of corresponding patients with RVO or RAO. Demographic information, medical history, and ophthalmic history were collected from the day of diagnosis of vascular occlusion (in the vascular occlusion study groups) and the age of matching (in the comparison group) up until the day of data extraction for all study groups. Patients with hemi-retinal vein occlusion (HRVO) were grouped with patients diagnosed with central RVO (CRVO) [29].

We included a broad range of dementia types to study the association between vascular occlusions and all-cause dementia, in line with previous reports [17,19]. The presence of dementia was defined by a diagnosis with at least one of the following ICD-10 codes and confirmed during chart review: F01 (vascular dementia), F02 (dementia in other diseases classified elsewhere), F03 (unspecified dementia), G10 (Huntington’s disease), G20 (Parkinson’s disease), G23 (other degenerative diseases of the basal ganglia), G30.1 (Alzheimer’s disease with late onset), G30.8 (other Alzheimer’s disease), G31.0 (frontotemporal dementia), and G31.83 (dementia with Lewy bodies). These diagnostic codes were assigned by a non-ophthalmologist, such as a neurologist or geriatrician, and were confirmed from a review of chart notes from the electronic medical record.

### 2.2. Study Variables

The following variables were collected: date of birth, self-reported race, sex, self-reported ethnicity, any medical history of diabetes mellitus, hypertension, hyperlipidemia, stroke, coronary heart disease, and dementia, as well as any ophthalmic history of diabetic retinopathy (DR), age-related macular degeneration (AMD), or glaucoma. Coronary heart disease (CHD) included any history of myocardial ischemia, angina, coronary atherosclerosis, coronary artery disease, coronary artery bypass graft, or coronary angioplasty. If dementia was present, we also recorded the date of dementia diagnosis and the type of dementia. The type, laterality, and date of diagnosis of retinal vascular occlusion were additionally collected for patients with retinal vascular occlusions.

### 2.3. Statistical Analysis

Statistical analysis was conducted using SAS version 9.4 and advised by a biostatistician (HJC). Baseline clinical and demographic characteristics were compared between patients with and without retinal vascular occlusions using the independent samples *t*-test for continuous variables and Pearson’s chi-square test for categorical variables. Continuous variables were presented as mean ± standard deviation (SD) and categorical variables were presented as percentages.

Primary outcomes included the presence of dementia and among those who had dementia, the age at dementia diagnosis and the type of dementia. Multivariate logistic regression was used to determine the association between (1) the presence of dementia and (2) vascular occlusions separated by type of occlusion (2a: artery versus vein) and subtype of occlusion (2b: branch versus central). Further multivariate logistic regression analysis was conducted looking at Alzheimer’s disease and vascular dementia as primary outcomes since they are the two most prevalent causes of dementia in this study and nationwide [30]. Multivariate linear regression was used to determine the association between the age at dementia diagnosis and the presence of vascular occlusion. All analyses were controlled for demographic and clinical covariates, including race, ethnicity, diabetes, hypertension, hyperlipidemia, stroke, and CHD, as well as eye diseases with a known association with AD, including DR, AMD, and glaucoma [15]. Odds ratios (ORs) and 95% confidence intervals (CIs) were presented for the presence of dementia and the type of dementia while mean ± SD was presented for the age at dementia diagnosis. *p*-values less than 0.05 were considered statistically significant.

## 3. Results

Our institution’s clinical data warehouse identified 718 patients above the age of 50 with retinal vascular occlusion using ICD-10 codes, but only 474 patients were confirmed to have a retinal vein or artery occlusion during chart review. Among these 474 patients, 34 patients had a dementia diagnosis based on ICD-10 code criteria. However, upon chart review, 45 patients with retinal vascular occlusion were found to have dementia.

Data from 474 patients with retinal vascular occlusions and 948 patients without retinal vascular occlusion (comparison group) were included in this study. Those with vascular occlusions included 413 patients with RVO (87.1%) and 61 patients with RAO (12.9%). Due to the age and sex matching, the two groups were similar with respect to age and sex (Table 1). The two groups were significantly different with respect to race and ethnicity. The study groups were also significantly different with respect to the following systemic and ophthalmic comorbidities: hypertension, hyperlipidemia, stroke, CHD, glaucoma, and DR. The distribution of age per study group is displayed in Table 2. The majority of patients in each study group were older than 70 years of age. Among patients with and without RVO (n = 1239), the prevalence of dementia (n = 86) was 6.9% (95% CI: 5.6% to 8.4%). Among patients with and without RAO (n = 183), the prevalence of dementia (n = 24) was 13.1% (95% CI: 8.2% to 18.0%). 

### 3.1. Presence of All-Cause Dementia in the Matched Vascular Occlusion–Comparison Analysis

Table 3 displays the association between retinal vein and arterial occlusion and the odds of having all-cause dementia. There were significantly increased odds of all-cause dementia (OR = 2.32; *p* < 0.001) in the RVO group relative to the comparison group. When stratifying by the type of RVO, there were significantly increased odds of having all-cause dementia in the CRVO group (OR = 2.32; *p* = 0.014) and in the BRVO group (OR = 2.68; *p* = 0.007) relative to their respective comparison groups. There were no significantly increased odds of the presence of dementia (OR = 1.01; *p* = 0.983) in the RAO group relative to the comparison group.

Stroke (OR = 2.63 (95% CI: 1.40–4.94); *p* = 0.003), CHD (2.58 (95% CI: 1.46–4.57); *p* = 0.001), glaucoma (OR = 1.74 (1.08–2.78); *p* = 0.022), and race (Black vs. White (OR = 1.85 (1.02–3.36); *p* = 0.043) and Other vs. White (0.15 (0.03–0.72); *p* = 0.017)) were significantly associated with all-cause dementia among patients with RVO and their corresponding comparison group patients. CHD (OR = 3.21 (95% CI: 1.03–9.95); *p* = 0.044) was significantly associated with all-cause dementia among patients with RAO and their corresponding comparison group patients. Of note, all of the aforementioned comorbidities were adjusted for as covariates in our multivariate model on the odds of all-cause dementia among those with retinal vein and artery occlusion.

Given the significant difference in race distribution between patients with and without vascular occlusions (Table 1) and the significant association between race and all-cause dementia, we tested the association between all-cause dementia and the interaction between race and the presence of vascular occlusion subtypes in a multivariate logistic regression adjusted for demographic, clinical, and ophthalmic covariates. There was no significant association between all-cause dementia and the interaction between race and RVO (*p* = 0.319) or RAO (*p* = 0.988).

### 3.2. Presence of AD or VD

The most frequent types of dementia were AD and VD in both the vascular occlusion and comparison groups (Table 4). There were significantly higher odds of the presence of VD in the RVO group (OR = 3.29; *p* = 0.006) relative to the comparison group (Table 5), but not in the RAO group (OR = 1.54; *p* = 0.663). There was no significant difference in the odds of the presence of AD in the RVO group (OR = 1.74; *p* = 0.133) or in the RAO group (OR = 0.32; *p* = 0.244) relative to their respective comparison groups. Other dementia subtypes and vascular occlusion subtypes were not tested due to a smaller sample size.

### 3.3. Age at Diagnosis of All-Cause Dementia

We did not find any association between a history of retinal vascular occlusions and the age at dementia diagnosis between patients with (80.76 ± 7.87 years) and without (79.86 ± 9.84 years; *p* = 0.434) RVO, or between patients with (83.11 ± 8.91 years) and without (80.48 ± 6.73 years; *p* = 0.507) RAO.

## 4. Discussion

Many diseases that affect the central nervous system can affect the eye, and the ophthalmologist is often the first to diagnose them [31,32,33,34,35]. The eye and the central nervous system share embryological origins, and some eye diseases including cataracts, glaucoma, and macular degeneration share common risk factors to Alzheimer’s disease in correlation studies.

This retrospective study found a significant association between retinal vein occlusions, both central and branch, and all-cause dementia. Our findings used an in-depth medical record review to support previously published population-based cohort studies [16,17,18,19,20], most of which relied on unconfirmed ICD-10 coding which can be limited by coding errors by clinicians [36,37]. For example, during our review, we found that 66% of patients identified as having an ICD-10 code for retinal vascular occlusion diagnosis were confirmed to have had an RVO or RAO; additionally, 24% of patients with confirmed dementia in the vascular occlusion group did not have an ICD-10 code for dementia listed in their chart. Our study also shows that the association between retinal vascular occlusion and dementia is likely driven primarily by retinal vein occlusions, with both CRVO and BRVO having higher odds for dementia, and a limited to no effect from retinal artery occlusions. Notably, we found that patients with retinal vein occlusions have higher odds of vascular dementia specifically, which was also not seen with retinal artery occlusions. Additionally, race appears to be independently associated with dementia in our study.

Retinal vascular diseases, specifically retinal vein occlusions, may reflect cerebrovascular disease and thus increase the risk of both AD and VD. The retinal imaging of patients with RVO and patients with AD show reduced blood flow [38,39], vessel density [40,41,42,43,44,45], perfusion density [45,46], and venule caliber [47,48], as well as an increased foveal avascular zone area [43,45]. The authors of the Rotterdam Scan Study also suggest that retinal venular dilatation may parallel cerebral venular obstruction and dilatation [49]. RVO involves the thickening of the retinal artery that compresses the adjacent retinal vein, leading to thrombus formation and vein occlusion [21]. As proposed by Lee et al., the thickening of the retinal artery could reflect similar changes to the cerebral vasculature in cerebral small vessel disease and intracranial atherosclerosis, both of which are associated with dementia [16,50,51]. Previous studies have found that cerebral small-vessel disease, which shares genetic risk factors with AD and may be involved in the pathogenesis of both AD and VD, is more frequent in patients with RVO and patients with retinal arterial narrowing and sclerosis [50,52,53]. Furthermore, visual impairment from RVO can be associated with social isolation which is a known risk factor for dementia [54,55].

Risk factors for RAO include smoking, hypertension, obesity, diabetes, hyperlipidemia, and cardiovascular disease [22]. These comorbidities also serve as risk factors for stroke, which is strongly associated with dementia [22]. Despite these risk factors, we did not observe a significant association between RAO and dementia in our study. This is consistent with a recent population-based cohort study published by Clausen et al., based on unconfirmed ICD-10 codes, reporting no significant association between retinal arterial occlusions and all-cause dementia, including AD and VD [19]. While we had fewer patients with RAO (n = 61) than patients with RVO (n = 413), the adjusted odds ratio for the association between retinal artery occlusion and all-cause dementia was approximately 1 and not statistically significant, indicating that the lack of a significant association of RAO and dementia is likely not due only to sample size. Overall, there appears to be a stronger association between dementia and RVO compared to dementia and RAO, and the reasons are unclear but likely due to differences in the pathophysiology of RVO and RAO. RAO is due to the arterial blockage of blood flow to the retina whereas RVO is due to thrombus occlusion at the lamina cribrosa or arteriovenous crossing [21,22].

In concordance with prior research, we found stroke and coronary heart disease to also be independently associated with all-cause dementia [56]. These comorbidities, as well as other systemic and eye comorbidities, such as glaucoma, DR, and AMD, were adjusted for in our model. In addition, rates of hypertension, hyperlipidemia, stroke, and coronary heart disease were significantly higher in the comparison group that did not develop RVO or RAO. While the reasons for the existence of higher rates of comorbidities in our comparison group are unclear, our results showing that patients with RVO having higher odds of dementia despite fewer comorbidities indicate that the association may be driven by factors other than these systemic conditions. Our baseline demographic characteristics show a significant difference in race distribution between patients with and without vascular occlusions (Table 1), but further analyses showed no significant association between dementia and the interaction between race and vascular occlusion, indicating that the association between retinal vascular occlusion and all-cause dementia is likely not significantly impacted by race in our cohort. That being said, the impact of race as an independent and unmodifiable risk factor of dementia warrants further investigation, including the effect of socioeconomic factors, such as low income and education, that are also associated with the development of dementia and may underlie the correlations that we found with race in our study [57,58].

This study has several strengths. This is the first retrospective in-depth chart review investigating the risk factors underlying the association between retinal vascular occlusions and dementia. Although Chan et al. briefly mention a record review, the diagnoses were identified by ICD-9 or ICD-10 codes and there is no mention of whether they were confirmed by a review of individual medical records, especially given the large number of participants. In our study, the validity of diagnostic codes was not a limiting factor because each chart of an included patient underwent an in-depth medical record review to confirm the ICD-10 code diagnoses and individual data points on medical and ophthalmic history, allowing us to adjust for additional confirmed comorbidities such as hyperlipidemia and coronary heart diseases as well as ophthalmic conditions including DR, AMD, and glaucoma. In addition, our study population was racially and ethnically diverse. Previous reports have shown that patients who identify as Black have been historically underrepresented in clinical trials for both dementia and eye disease [59,60,61,62], and previous studies investigating the association between retinal vascular occlusions and dementia were large-scale population-based cohorts with limited diversity or with an unknown demographic distribution [36,37].

Our study is limited by its retrospective study design, small sample size, potential for selection bias, and external validity restrictions. Given that this is a cross-sectional study evaluating the odds of the presence of dementia rather than the longitudinal development or progression of dementia, we did not control for loss to follow up or death. Since our objective was to demonstrate shared underlying risk factors and not establish a causal relationship, we did not include a survival analysis. Additionally, we could not account for genetic susceptibility genes, such as apolipoprotein E, with an established role in the development of dementia. Furthermore, the patients included in this study were registered patients of the eye clinic such that the analyses could adjust for the presence of eye diseases including DR, AMD, and glaucoma. However, this may have led to selection bias since patients of the eye clinic would have a higher proclivity for eye diseases, including those associated with dementia, compared to the general patient population seen at our hospital. This may limit the external validity of our study. On the other hand, patients with eye disease represent an at-risk population for dementia and are therefore a potentially worthy target population for study. Additionally, our study groups significantly differed in terms of several demographic characteristics (Table 1), including stroke, CHD, race, and glaucoma. The presence of these comorbidities may have affected the association between retinal vascular occlusion and dementia, but we adjusted for these factors in the multivariable analysis to mitigate any confounding effects. However, stroke, CHD, and glaucoma share underlying risk factors, so an issue with multicollinearity remains and undermines the significant relationship between RVO and all-cause dementia. On the other hand, hypertension, hyperlipidemia, stroke, and CHD were seen at higher rates in the comparison group without retinal vascular occlusion, which indicates that the significant associations seen between RVO and dementia were related to factors other than these comorbidities. Additionally, fewer than 50 participants per study group were diagnosed with all-cause dementia, AD, or VD (Table 4), indicating that our analyses may be vulnerable to small-sample bias. The small-sample bias may account for the lack of significant associations between retinal vein occlusions and Alzheimer’s disease [18]. In our study, a limited sample size likely impacted the vein occlusion and AD analysis; the odds ratio for the association between RVO and AD was nearly 2, yet not significant, indicating that our cohort may have been underpowered. Lastly, there is a potential for confounding from uncontrolled factors that were not recorded in a standardized manner, such as smoking status, the severity of comorbidities, and medication history.

## 5. Conclusions

In conclusion, our study confirms the eye’s strong connection to the brain with our findings that both branch retinal and central retinal vein occlusions, but not arterial occlusions, appear to be independently associated with all-cause dementia and vascular dementia. Clinicians should be aware that patients with RVO are at increased risk of co-existing or eventual dementia, and that these patients may also benefit from referral to primary care or neurology for initial evaluation and ongoing monitoring for dementia and other neurocognitive disorders. Future steps may include an investigation into the role of retinal arterial occlusions, the impact of genetic susceptibility, and the molecular mechanism of CRVO and BRVO as they relate to the development of dementia.

## Figures and Tables

**Table 1:** Baseline demographic characteristics of patients with and without (comparison group) retinal vascular occlusions.

	Retinal Vein Occlusion (RVO) Group (n = 413)	Retinal Artery Occlusion (RAO) Group (n = 61)	Comparison Group (n = 948)	*p*-Value
Age (years)	74.40 ± 11.70	75.59 ± 11.79	74.45 ± 11.00	0.733
Sex				0.999
Female	211 (51.1%)	34 (55.7%)	490 (51.7%)	
Male	202 (48.9%)	27 (44.3%)	458 (48.3%)	
Race				<0.001
Black	188 (45.5%)	25 (41.0%)	670 (70.7%)	
White	96 (23.2%)	18 (29.5%)	234 (24.7%)	
Other	129 (31.2%)	18 (29.5%)	44 (4.6%)	
Ethnicity				<0.001
Hispanic/Latino	74 (17.9%)	13 (21.3%)	42 (4.4%)	
Not Hispanic/Latino	339 (82.1%)	48 (78.7%)	906 (95.6%)	
Diabetes	157 (38.0%)	21 (34.4%)	408 (43.0%)	0.074
Hypertension	262 (63.4%)	31 (50.8%)	655 (69.1%)	0.003
Hyperlipidemia	108 (26.2%)	22 (36.1%)	422 (44.5%)	<0.001
Stroke	21 (5.1%)	8 (13.1%)	87 (9.2%)	0.014
Coronary Heart Disease	38 (9.2%)	12 (19.7%)	140 (14.8%)	0.007
Glaucoma	132 (32.0%)	4 (6.6%)	217 (22.9%)	<0.001
Age-related Macular Degeneration	23 (5.6%)	1 (1.6%)	30 (3.2%)	0.068
Diabetic Retinopathy	73 (17.7%)	8 (13.1%)	102 (10.8%)	0.002

**Table 2:** Distribution of the age of patients included in each study group. The majority of patients in each study group was older than 70 years of age.

Age Group (Years)	RVO (n = 413) (n and %)	RAO (n = 61) (n and %)	Comparison Group (n = 948) (n and %)
50–59	52 (12.6%)	7 (11.5%)	112 (11.8%)
60–69	99 (24.0%)	14 (23.0%)	247 (26.1%)
70–79	122 (29.5%)	16 (26.2%)	299 (31.5%)
80–89	102 (24.7%)	19 (31.1%)	220 (23.2%)
90–99	31 (7.5%)	5 (8.2%)	72 (7.6%)
100+	7 (1.7%)	0 (0%)	8 (0.8%)

**Table 3:** Odds ratios for all-cause dementia after adjusting for demographic, medical, and ophthalmic covariates in the matched vascular occlusion–comparison analysis. A multivariate logistic regression model was used to identify odds for the presence of all-cause dementia. Covariates included race, ethnicity, diabetes, hypertension, hyperlipidemia, stroke, coronary heart disease, diabetic retinopathy, age-related macular degeneration, and glaucoma. *p*-values less than 0.05 were considered statistically significant and are bolded.

	N of Group	N and % of Dementia Cases	Adjusted Odds Ratio	Adjusted 95% CI	*p*-Value
Retinal Vein Occlusion (RVO)					
RVO Group	413	38 (9.2%)	2.32	1.44–3.75	**<0.001**
Comparison Group	826	48 (5.8%)			
Central Retinal Vein Occlusion (CRVO)					
CRVO Group	192	21 (10.9%)	2.32	1.19–4.54	**0.014**
Comparison Group	384	27 (7.0%)			
Branch Retinal Vein Occlusion (BRVO)					
BRVO Group	221	17 (7.7%)	2.68	1.30–5.50	**0.007**
Comparison Group	442	21 (4.8%)			
Retinal Artery Occlusion (RAO)					
RAO Group	61	7 (11.5%)	1.01	0.32–3.26	0.983
Comparison Group	122	17 (13.9%)			

**Table 4:** Types of dementia in the retinal vein occlusion (RVO), retinal artery occlusion (RAO), and comparison study groups.

	RVO Group (n = 413)	RAO Group (n = 61)	Comparison Group (n = 948)
All-cause dementia	38 (9.2%)	7 (11.5%)	65 (6.9%)
Alzheimer’s disease	14 (3.4%)	2 (3.3%)	32 (3.4%)
Vascular dementia	12 (2.9%)	3 (4.9%)	19 (2.0%)
Mixed AD and VD	1 (0.2%)	0 (0%)	5 (0.5%)
Parkinson’s disease dementia	2 (0.5%)	2 (3.3%)	3 (0.3%)
Lewy body dementia	1 (0.2%)	0 (0%)	1 (0.1%)
Unspecified dementia	8 (1.9%)	0 (0%)	5 (0.5%)

**Table 5:** Odds ratios for the presence of Alzheimer’s disease (AD) and vascular dementia (VD) among patients with and without retinal vein (RVO) or arterial (RAO) occlusions. The analysis involved a multivariate logistic regression test. *p*-values less than 0.05 were considered statistically significant and are bolded.

	N of Group	Alzheimer’s Disease (AD)			Vascular Dementia (VD)		
		Number and % of AD Cases	Adjusted Odds Ratio (95% Confidence Interval)	*p*-Value	Number and % of VD Cases	Adjusted Odds Ratio (95% Confidence Interval)	*p*-Value
Retinal Vein Occlusion (RVO)							
RVO Group	413	14 (3.4%)	1.74 (0.84–3.59)	0.133	12 (2.9%)	3.29 (1.41–7.68)	**0.006**
Comparison Group	826	21 (2.5%)			14 (1.7%)		
Retinal Artery Occlusion (RAO)							
RAO Group	61	2 (3.3%)	0.32 (0.05–2.20)	0.244	3 (4.9%)	1.54 (0.22–10.81)	0.663
Comparison Group	122	11 (9.0%)			5 (4.1%)		

## Data Availability

The data presented in this study are available on request from the corresponding author due to privacy or ethical restrictions.
